# Machine-learning analysis of cross-study samples according to the gut microbiome in 12 infant cohorts

**DOI:** 10.1128/msystems.00364-23

**Published:** 2023-10-24

**Authors:** Petri Vänni, Mysore V. Tejesvi, Niko Paalanne, Kjersti Aagaard, Gail Ackermann, Carlos A. Camargo, Merete Eggesbø, Kohei Hasegawa, Anne G. Hoen, Margaret R. Karagas, Kaija-Leena Kolho, Martin F. Laursen, Johnny Ludvigsson, Juliette Madan, Dennis Ownby, Catherine Stanton, Jakob Stokholm, Terhi Tapiainen

**Affiliations:** 1 Research Unit of Clinical Medicine, University of Oulu, Oulu, Finland; 2 Ecology and Genetics, Faculty of Science, University of Oulu, Oulu, Finland; 3 Department of Pediatrics and Adolescent Medicine, Oulu University Hospital, University of Oulu, Oulu, Finland; 4 Department of Obstetrics & Gynecology, Division of Maternal-Fetal Medicine, Baylor College of Medicine and Texas Children’s Hospital, Houston, Texas, USA; 5 Department of Pediatrics, University of California, San Diego, California, USA; 6 Department of Emergency Medicine, Massachusetts General Hospital, Harvard Medical School, Boston, Massachusetts, USA; 7 Department of Climate and Environmental Health, Norwegian Institute of Public Health, Oslo, Norway; 8 Department of Clinical and Molecular Medicine, Norwegian University of Science and Technology, Trondheim, Norway; 9 Department of Epidemiology, Geisel School of Medicine, Dartmouth College, Hanover, New Hampshire, USA; 10 Children’s Hospital, University of Helsinki and HUS, Helsinki, Finland; 11 National Food Institute, Technical University of Denmark, Lyngby, Denmark; 12 Crown Princess Victoria Children’s Hospital and Division of Pediatrics, Department of Biomedical and Clinical Sciences, Linköping University, Linköping, Sweden; 13 Department of Psychiatry, Dartmouth Hitchcock Medical Center, Geisel School of Medicine at Dartmouth, Lebanon, New Hampshire, USA; 14 Department of Pediatrics, Dartmouth Hitchcock Medical Center, Geisel School of Medicine at Dartmouth, Lebanon, New Hampshire, USA; 15 Medical College of Georgia, Augusta, Georgia, USA; 16 Teagasc Food Research Centre & APC Microbiome Ireland, Moorepark, Fermoy, Co. Cork, Ireland; 17 Herlev and Gentofte Hospital, University of Copenhagen, Copenhagen, Denmark; 18 Department of Food Science, University of Copenhagen, Copenhagen, Denmark; 19 Biocenter Oulu, University of Oulu, Oulu, Finland; University of California, San Francisco, California, USA

**Keywords:** machine learning, bioinformatics, human microbiome, gut microbiome, random forest, infant, children, cross-study, ensemble

## Abstract

Combining and comparing microbiome data from distinct infant cohorts has been challenging because such data are inherently multidimensional and complex. Here, we used an ensemble of machine-learning (ML) models and studied 16S rRNA amplicon sequencing data from 4,099 gut microbiome samples representing 12 prospectively collected infant cohorts. We chose the childbirth delivery mode as a starting point for such analysis because it has previously been associated with alterations in the gut microbiome in infants. In cross-study ensemble models, Bacteroides was the most important feature in all machine-learning models. The predictive capacity by taxonomy varied with age. At the age of 1–2 months, gut microbiome data were able to predict delivery mode with an area under the curve of 0.72 to 0.83. In contrast, ML models trained on taxa were not able to differentiate between the modes of delivery, in any of the cohorts, when the infants were between 3 and 12 months of age. Moreover, no ML model, alternately trained on the functional pathways of the infant gut microbiome, could consistently predict mode of delivery at any infant age. This study shows that infant gut microbiome data sets can be effectively combined with the application of ML analysis across different study populations.

**IMPORTANCE:**

There are challenges in merging microbiome data from diverse research groups due to the intricate and multifaceted nature of such data. To address this, we utilized a combination of machine-learning (ML) models to analyze 16S sequencing data from a substantial set of gut microbiome samples, sourced from 12 distinct infant cohorts that were gathered prospectively. Our initial focus was on the mode of delivery due to its prior association with changes in infant gut microbiomes. Through ML analysis, we demonstrated the effective merging and comparison of various gut microbiome data sets, facilitating the identification of robust microbiome biomarkers applicable across varied study populations.

## INTRODUCTION

It has been suggested that childbirth delivery mode, Caesarean delivery, is associated with varying degrees of greater risk of non-communicable diseases later in life, notably asthma (1, 2), food allergies (3, 4), obesity (5), and diabetes (6) among offspring even though studies with high-quality designs have not given consistent results regarding these associations (7). In most studies, but not all, Caesarean delivery has been associated with an altered gut microbiome composition for neonates or infants (8) principally relatively lower abundances of *Escherichia-Shigella* (9) and *Parabacteroide*s (10) and delayed colonization with *Bacteroides* (9–14) and *Bifidobacterium* (10, 14, 15) with a contrasting relative enrichment in *Clostridium* (10, 11)

Most gut microbiome studies have used sequencing data of the bacterial 16S rRNA gene or less often whole-genome bacterial sequencing. As microbiome data are multidimensional and noisy, it is difficult to combine data from two or more populations for traditional statistical testing of a hypothesis (16). It has been suggested that machine learning (ML) models may help to overcome this limitation (17–19) because the model can train on specific data set and then be used on further data set, and its efficiency validated. To date, there have been a limited number of studies combining or comparing gut microbiome data from several available prospective cohort studies in neonates, infants, or children using the ML approach, such as random forest (17–20), support vector machine (17, 18), elastic net (17, 18), and gradient-boosted machine algorithms (18).

Here, we use an ensemble of ML models, including random forest (21) (RF), extremely randomized trees (22) (EXTRA), light gradient-boosting machine (23) (LGBM), and multilayer perceptron (MLP) predictive models across 12 prospective pediatric cohorts with gut microbiome data originating from 6 different countries. To evaluate the usefulness of ML algorithms in combining and comparing microbiome data across different cohort studies, we compared the association of delivery mode with gut microbiome composition in the cohorts.

## MATERIALS AND METHODS

### Literature search and data set recruitment

**Fig 1:**
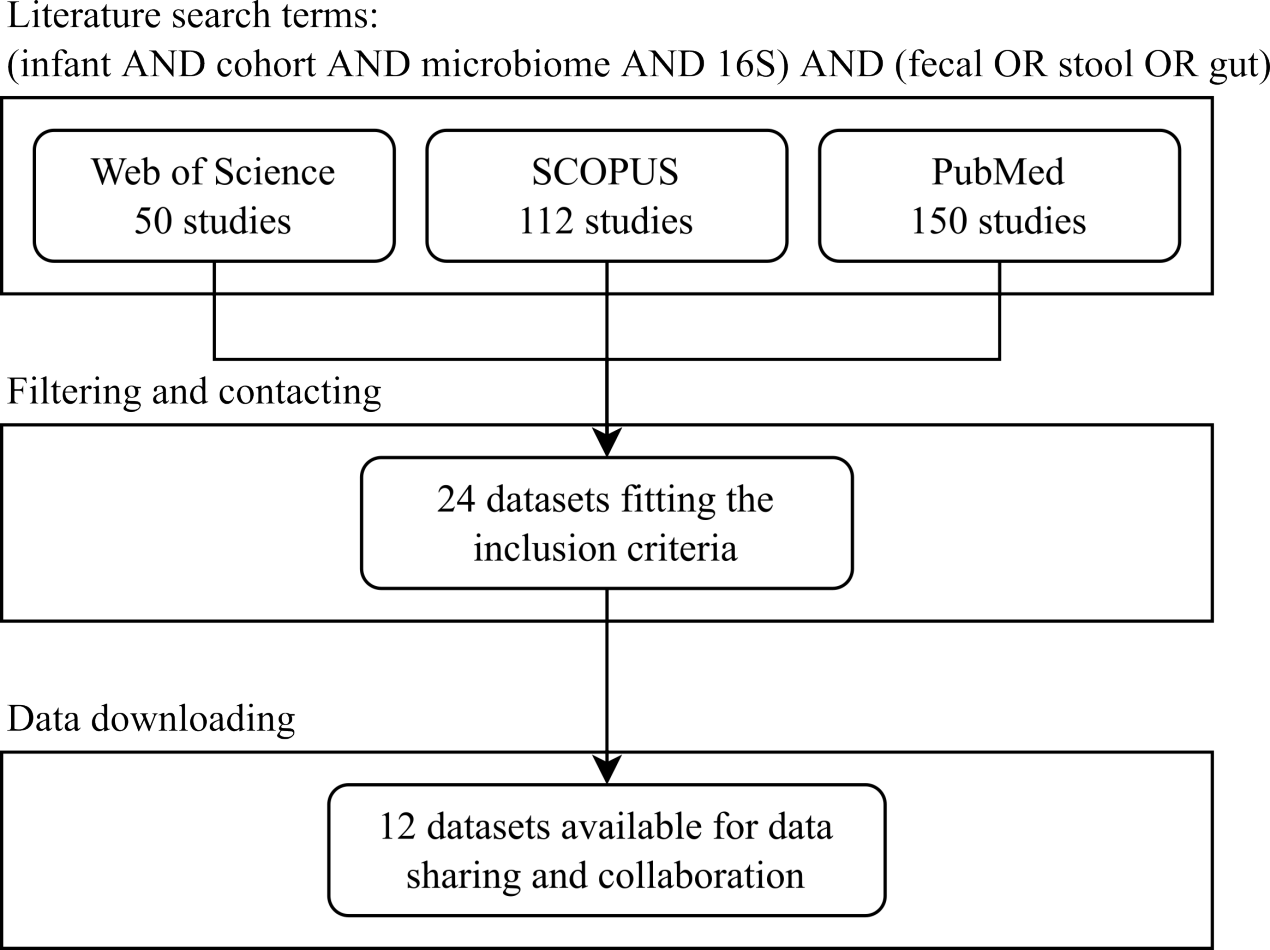
Flowchart of the literature search.

A systematic literature review was conducted in the Web of Science, Scopus, PubMed, and Google Scholar databases up to January 2020. Additionally, the clinicaltrials.gov website was searched for suitable studies (Fig. 1). We used the following terms to search through the titles, abstracts, and keywords of the literature in our set of materials: (infant AND cohort AND microbiome AND 16S) AND (fecal OR stool OR gut). Our inclusion criteria for data sets were that the studies should have more than 50 infant fecal samples with 16S microbiome data available from the first 12 months of life, with defined sampling times. Birth cohorts containing only preterm infants were excluded. The data set correspondents were invited to participate in this multicohort collaboration.

All the institutions’ original data sets and protocols were approved by their institutional review boards and ethical committees, and all families of the infants provided their written informed consent. Only 16S rRNA amplicon sequence data and data on the delivery mode and breastfeeding were used here, and no individual personal data were transferred or used.

After contacting 24 research groups, 12 of these groups participated and provided access to 16S rRNA amplicon sequence data sets of fecal samples, along with data on the mode of delivery (Table 1). Microbiome development causes large shifts over time in the gut microbiome of infants in the first year of life (24, 25). As such, we analyzed data sets in three age groups: 1–2 months, 3–6 months, and 9–12 months. Seven infant cohorts had fecal samples available 1–2 months after birth, four cohorts had samples 3–6 months after birth, and eight had samples 9–12 months after birth. Altogether, we had 16S rRNA amplicon sequencing data available for 4,099 fecal samples, or 3,595 samples, after pre-processing and quality filtering. There were 1,457 samples collected at 1–2 months of age, of which 440 were from infants delivered by Caesarean and 1,017 were from infants delivered vaginally (Table 1). At 3–6 months of age, we had 473 samples, comprising 201 from infants delivered by Caesarean and 272 samples from vaginally delivered infants. At 9–12 months of age, we had 1,665 samples, of which 363 were from infants delivered by Caesarean and 1,302 were from infants delivered vaginally.

**TABLE 1:** Study cohort characteristics

	Country of origin	Initial number of fecal samples	Available samples after pre-processing	Infants born via Caesarean delivery	Infants born via vaginal delivery	Mean age of infants (months)
Sampled at 1–2 months						
COPSAC (26)	Denmark	505	303	72	231	1
HOUSTON^a^(27)	USA	52	48	11	37	1.5
INFANTMET (28)	Ireland	167	137	65	72	1
JORVI (29)	Finland	68	51	8	43	1
NHBCS (30)	USA	321	319	92	227	1.5
NOMIC (31)	Norway	485	485	159	326	1
WHEALS (32)	USA	130	114	33	81	1.2
Sampled at 3–6 months						
INFANTMET (28)	Ireland	152	152	86	66	5.5
JORVI (29)	Finland	68	62	10	52	6
MARC-43 (33)	USA	115	115	43	72	3.4
WHEALS (32)	USA	167	144	62	82	6.6
Sampled at 9–12 months						
ABIS (34)	Sweden	403	399	47	352	12
COPSAC (26)	Denmark	623	424	90	334	12
JORVI (29)	Finland	62	62	10	52	12
NHBCS (30)	USA	135	135	38	97	12
NOMIC (31)	Norway	340	340	103	237	12
OULU (35)	Finland	84	84	23	61	12
SKOT1 (36)	Denmark	115	115	16	99	9
SKOT2 (36)	Denmark	107	106	36	70	9

^a^ Pregnant women prospectively enrolled in the early third trimester.

### Sequence pre-processing

**Fig 2:**
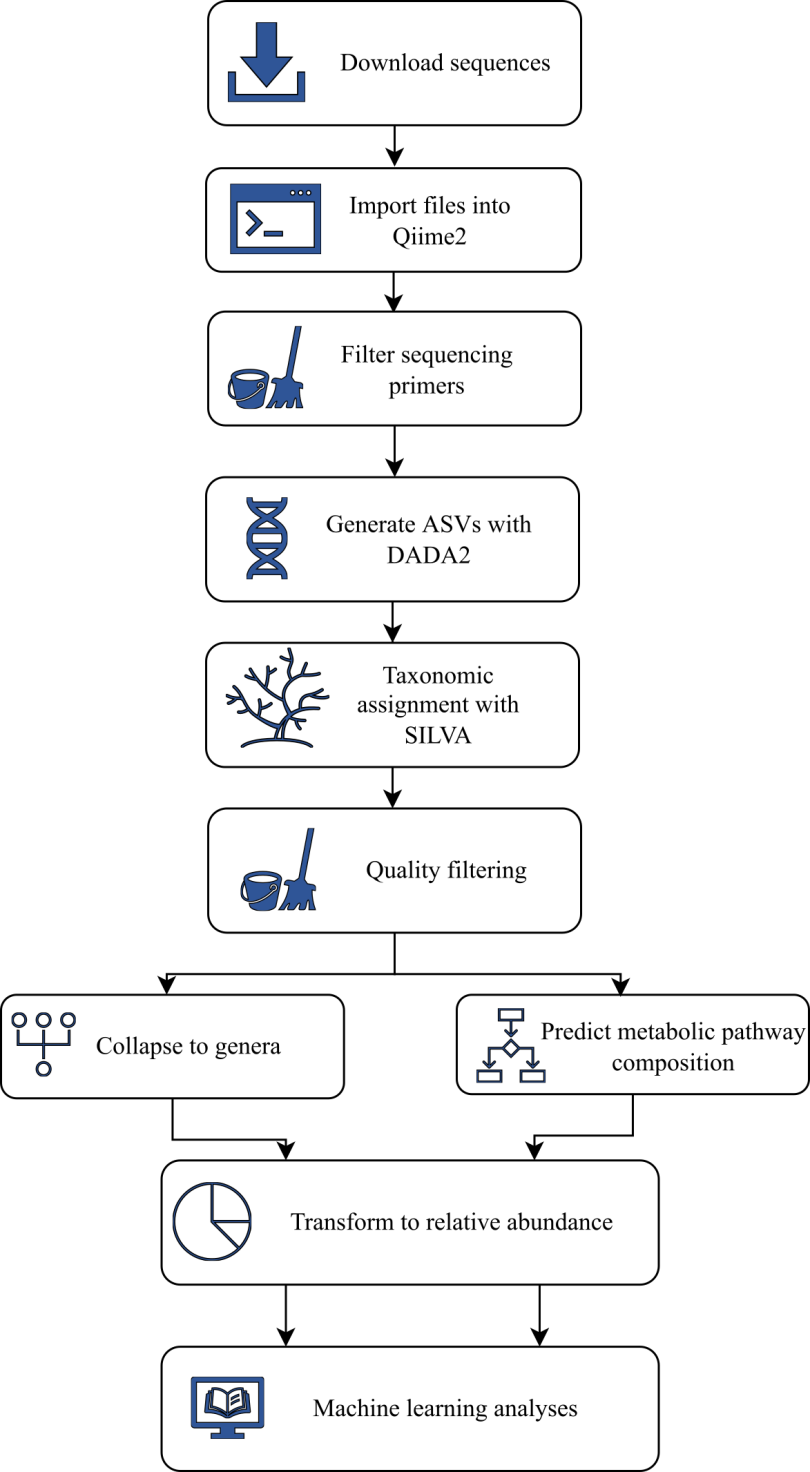
Step-by-step flowchart of the pre-processing pipeline.

Before the data were analyzed using ML methods, each data set was prepared, quality filtered using similar methods, transformed into relative abundance information for each bacterial taxon or metabolic pathway in each sample, and presented in feature tables. The pre-processing pipeline is shown in Fig. 2. The sequences were downloaded from their repositories or acquired directly from the corresponding researchers (Table 1) before being imported into the Qiime2 (37) (version 2021.11) microbiome bioinformatics platform using the q2-tools module. The primer sequences were removed from each data set using the q2-cutadapt tool, and the open-source software package DADA2 (38) was used to de-noise the sequences into amplicon sequence variants (ASVs) using the q2-dada2 module, where the trunc-len parameter was set to zero. ASVs, found in fewer than 2 samples and in a total frequency of 10, were removed. Taxonomy was assigned using the SILVA (39) (version 138) database with a Naïve Bayes classifier. ASVs classified as mitochondria or chloroplasts were removed. The ASVs were collapsed to the taxonomic level of genera and transformed into relative abundances for downstream analyses. A predicted metabolic pathway composition was produced from the filtered ASV feature table using PICRUSt2 software (40). Additionally, a second set of data sets was generated with Greengenes (41) instead of SILVA for alternative ML model-building attempts. Each data set was pre-processed in the same way, except for the data from NOMIC, which were only available as a pre-processed ASV table with the taxonomic classification assigned with only Greengenes (41) database. A representative sequence file was prepared, and the data for pre-processing were input into the pre-processing pipeline as a taxonomic assignment step (Fig. 2), after which the protocol continued as for the other data sets.

### Alpha and beta diversity of the microbiome between cohorts

Diversity indices were calculated from rarefied genera-collapsed feature tables to visualize cohort differences with the q2 diversity plugin. For each sampling interval, the rarefying depth was chosen with the following rules: (i) depth of 1,000 is the minimum and (ii) choose the next highest value without losing samples in the rarefying process. For the 1–2 months time period, the chosen depth was 1,027; for 3–6 months, it was 1,116; and for 9–12 months, it was 1,009. Cohort and sample diversity were analyzed with Shannon diversity index and Bray–Curtis dissimilarity index for the alpha and beta diversity, respectively. The Bray–Curtis dissimilarity index was further analyzed with PCoA. The results were plotted with Matplotlib (42), and Kruskal–Wallis H-tests were conducted to examine the statistical differences between infants born via Caesarean delivery and those born via vaginal delivery. *P*-values were adjusted for multiple testing using the Benjamini–Hochberg procedure.

### Machine-learning analyses

Before training an ML model, a set of settings, i.e., “hyperparameters,” needs to be defined, followed by a search for the optimal combination. These settings define the structure and behavior of the models, such as the depth of the decision trees or the number of layers in a neural network. A common way of tuning the hyperparameters is to use a nested cross-validation method, in which the hyperparameters are selected using the training fold with an additional cross-validation loop (43). Here, the ML models were trained, tested, and validated by means of a nested cross-validation scheme with 40 repetitions. Each data set was first split in an outer cross-validation loop, where each fold, in turn, was used as the validation fold and the rest of the data were used in an inner cross-validation loop. The model building and parameter tuning took place only in the inner cross-validation loop. The performance of the final model was validated using the outer cross-validation folds and then averaged and recorded. Previous microbiome studies have chosen different *k* values for *k*-fold cross validation, such as 5 (44) or 10 (17, 18) folds. Ten folds have been recommended for biomedical data with high dimensions (45). As such, to maximize number of samples used in model training, the number of folds was set to as close to 10 as possible. In both *k*-fold cross-validation loops, the number of folds was set at 10, except in the JORVI cohort (8 outer folds and 7 inner folds) and the HOUSTON cohort (10 outer folds and 9 inner folds) for fecal samples obtained at 1–2 months of age and the JORVI cohort (10 outer folds and 9 inner folds) for samples obtained at 3–6 and 9–12 months of age, as there must be at least one of each class (Caesarean delivery and vaginal delivery) in the testing and validation folds to calculate the receiver operating characteristic (ROC) curve.

The feature importance of the models was estimated using the scikit-learn (46) function termed permutation importance. The scikit function takes in a trained model and testing data set where each feature is shuffled among all the samples in the testing data. Feature importance is defined by how much the prediction performance of the model is lowered following the shuffling as compared with a situation in which the feature is included in the model. In brief, a higher feature importance value indicates a greater importance of the feature to the model.

The ML classifier performances were estimated using the area under the curve (AUC) for the ROC and precision-recall (PR) curves. The model performance and feature importance values were averaged over 40 nested cross-validation loop repetitions.

### Feeding mode analyses

To better understand our findings, we ran additional analyses on feeding mode in the cohorts for which we had feeding mode data available at the 1–2 months time point. With these *post hoc* analyses, we attempted to control for the confounding effect of breastfeeding when predicting delivery mode. We produced additional analyses using the 1–2 months data sets from COPSAC, HOUSTON, INFANTMET, and JORVI, where infants fed only formula were removed.

We also employed the Fisher’s exact test to examine if Caesarean delivery or vaginally delivered groups had statistically more exclusively breastfed, partially breastfed, or formula-fed only infants in each cohort where breastfeeding data were available.

### Machine-learning hyperparameter tuning

Since decision tree-based algorithms have performed well in previous microbiome studies (17, 18, 20, 47, 48) and because neural networks (47, 49) show great promise for the analysis of several microbiome-related problems, we chose three decision tree algorithms and one deep learning algorithm for use here: RF (21), EXTRA (22), MLP, and the LGBM (23). The hyperparameters were tuned in the inner cross-validation loop using the scikit-learn RandomizedSearchCV function in which the n_iter parameter was set at 40 iterations. RandomizedSearchCV was used to tune the hyperparameter efficiently without having to go through all possible hyperparameter iterations (50).

The hyperparameters tuned for the random forest and extremely randomized trees algorithms were max_depth, max_features, class_weight, and bootstrap, the last-mentioned for random forest only. The hyperparameters searched for LGBM models were num_leaves, max_depth, n_estimators, reg_alpha, and learning_rate. The MLP models were trained using the “adam” solver in scikit-learn, the hyperparameters that were tuned being max_iter, alpha, learning_rate_init, and momentum. In the hyperparameter “hidden_layer_sizes,” the number of layers ranged from 1 to 3, with 10, 30, 50, or 100 neurons in the first layer, while in the models with multiple layers, each subsequent layer had half the number of neurons than the previous layer. The MLP hyperparameters for parameter tuning were chosen based on previously published work (49). The exact hyperparameter values used for parameter tuning are shown in Table S1.

### Cross-study machine learning using gut microbiome data

We then used a cross-study approach in which we aimed to test whether an ML model developed using certain given data sets is generalizable to other data sets with regard to the mode of delivery as an explanatory variable for gut microbiome composition. In addition, the mode of delivery was predicted in a cross-study manner so that each cohort’s outer cross-validation samples were predicted using best-performing models for all the other cohorts. These models were collected into an ensemble classifier in which the delivery mode of a given validation sample was predicted based on the averaged prediction of the best models for all the other cohorts. In this way, the same testing samples can be used for both the within-study and cross-study methods, and the performances are more readily comparable.

### PipelineSearch and control augmenting methods

There are countless combinations and ways in which to build ML models, and these can produce different results. Similarly, there are several options for each preprocessing step when handling 16S sequencing samples that affect downstream analyses, such as which software, taxonomic database, or collapsing level to choose (51, 52). Therefore, we developed “PipelineSearch” as a novel method to automate those choices. Instead of the researcher choosing which taxonomic database to use, such as SILVA or Greengenes, PipelineSearch they are chosen at the same time as hyperparameters in ML parameter tuning. During hyperparameter tuning, the models could select which taxonomic database was used in preprocessing, Greengenes or SILVA. Similarly, PipelineSearch could select between feature table types, predicted metabolic pathways or genera collapsed data.

We also used an approach referred to as control augmenting (20), in which additional control samples from outside data sets were added to the training data for the models. Wirbel et al. (20) increased the number of control samples fivefold in each training fold. To achieve similar numbers, we considered COPSAC and INFANTMET the augmenting cohort, as they had the most control samples at their respective time points. We did not consider the NOMIC data set, as we had no control over its early pre-processing steps, and thus, in this approach, the additional control samples came from COPSAC (1–2 months group), INFANTMET (3–6 months group), and COPSAC (6–9 months group) for their respective sampling time points. The COPSAC and INFANTMET cohorts were not used for model building or cross-study validation at the sampling times, where they were used to augment all the other cohorts, as this would leak information between validation folds.

### Reproducibility and code availability

The code used in the present ML analyses is available in the GitHub repository (https://github.com/pvanni/PipelineSearch). We reported our finding according to the Strengthening The Organization and Reporting of Microbiome Studies guidelines (53) and the checklist can be found in the GitHub repository. Installed Python packages are listed in (Table S2).

Random number generators were seeded to guarantee identical outer cross-validation splits for each algorithm choice in addition to rendering the results reproducible. In this way, each model was validated using the same validation samples, making direct comparison of their AUC values reliable in both within-study and cross-study predictions.

Full sequencing data for all cohorts used can be found from public data repositories, and their corresponding accession numbers can be found in (Table S3). Relative abundance feature tables from all cohorts used in genera and predicted pathway ML-analyses can be found in the supplemental material (Data S1 through S4) with delivery mode metadata linked to each sample as the last column.

## RESULTS

### Characteristics of the cohorts

**Fig 3:**
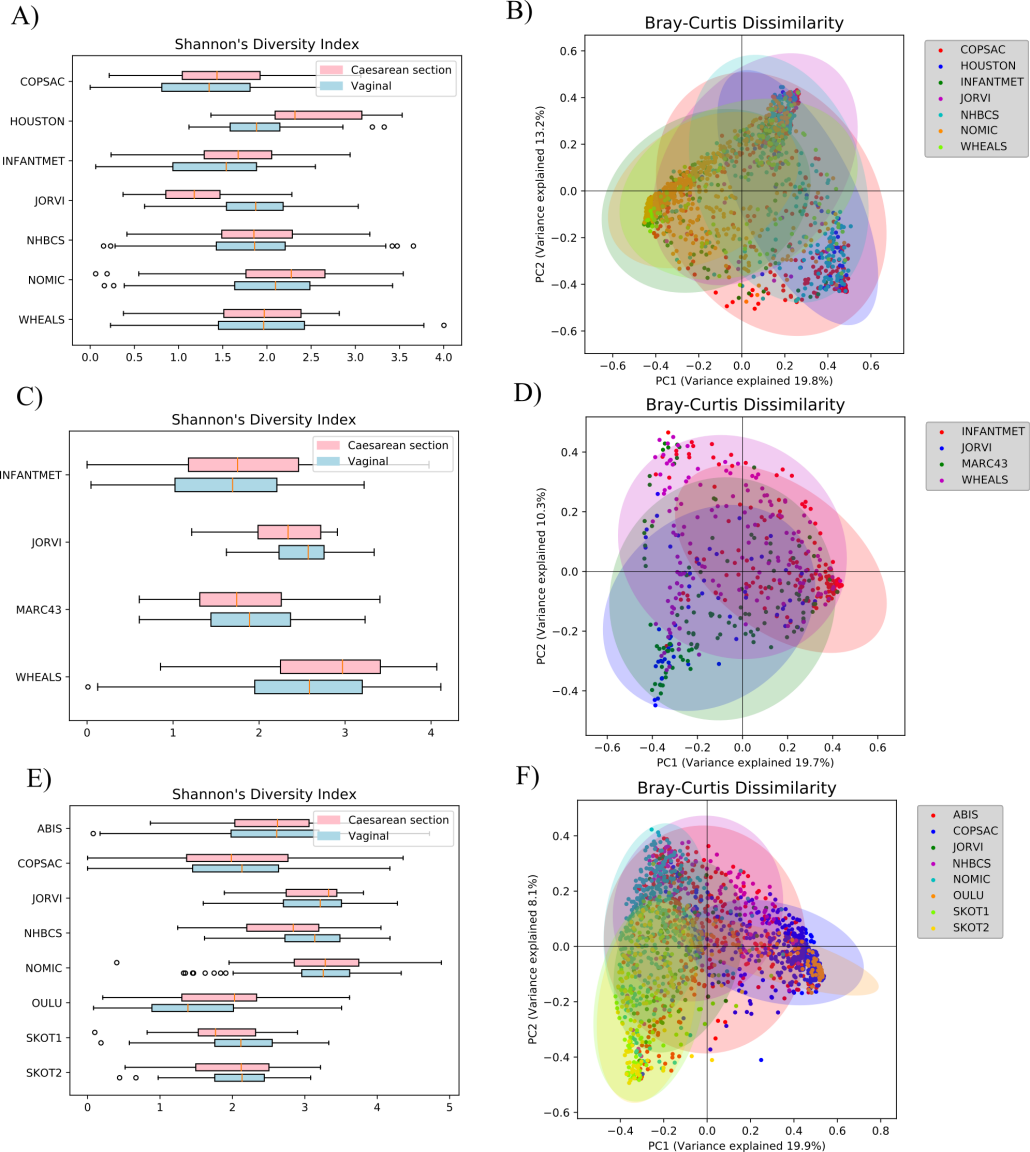
Alpha and beta diversity indices for each cohort. Within-sample diversity was analyzed using Shannon’s diversity indices for gut microbiomes sampled at (**A**) 1–2 months, (**C**) 3–6 months, and (**E**) 9–12 months, and the results were visualized with boxplots, where Caesarean delivery samples (pink) and vaginal delivery samples (light blue) were plotted separately for each cohort. Outliers detected by the plotting software were drawn as circles. Between-sample diversity was analyzed using Bray-Curtis dissimilarity at (**B**) 1–2 months, (**D**) 3–6 months, and (**F**) 6–9 months sampling time points using principal coordinate analysis (PCoA). The samples from each cohort were drawn in a different color within the sampling time points, and the confidence ellipse was drawn using the Pearson correlation coefficient for each cohort.

The general characteristics of the populations and the 16S rRNA amplicon sequence data sets are presented in Table 1 and (Table S3). The microbiome data sets were further characterized by plotting alpha and beta diversity indices for each infant cohort (Fig. 3) and for each time point (Fig. S1). Shannon’s diversity index was, on average, lower in the 1–2 months cohorts (mean = 1.8, SD = 0.26) than in the 3–6 months (mean = 2.16, SD = 0.37) or 9–12 months (mean = 2.46, SD = 0.58) cohorts. Alpha diversity did not differ significantly according to mode of delivery in any of the cohorts (Fig. 3). The Fisher’s exact test showed no significant enrichment of breast- or formula-fed samples in either Caesarean delivery or vaginally delivered groups in any cohort (Tables S4 and S5).

### The machine-learning models accurately predicted the delivery mode from the fecal microbiome taxonomic data at 1–2 months of age

**Fig 4:**
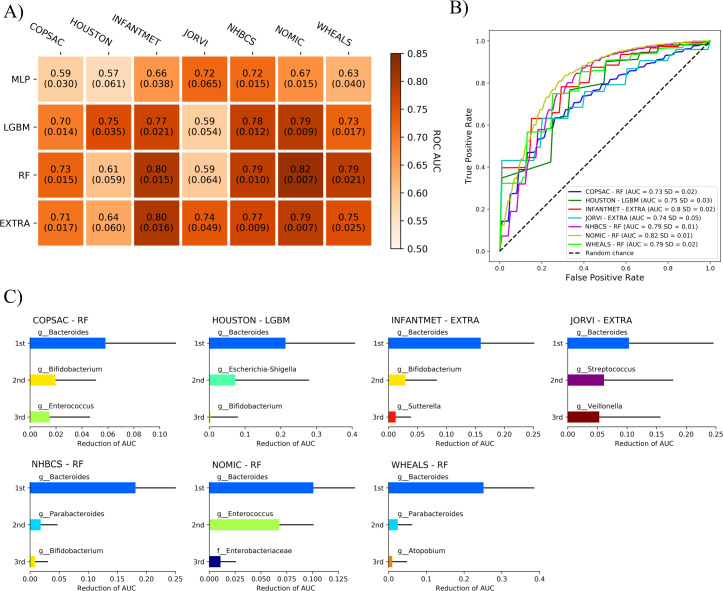
Machine-learning models can differentiate between the delivery modes of infants based on gut microbiome data at 1–2 months of age. The delivery modes were either vaginal delivery or Caesarean delivery, and the ML models used the gut microbiomes of the infants as assessed from fecal samples obtained approximately 1–2 months after birth in seven infant cohorts. (**A**) Best performances of the MLP, LGBM, RF, and EXTRA models, trained independently to differentiate between vaginal delivery and Caesarean delivery samples using the relative abundances of gut bacteria at 1–2 months after birth, shown separately for each cohort. (**B**) ROC curves for the best-performing models. The AUC values for these ROC curves indicate model performances that range between 0.5 and 1.0. Predictions from a model with a performance close to 0.5 are equivalent to a random choice, whereas a model with an AUC of 1.0 would hypothetically be a perfect model and classify all children correctly. (**C**) Permutation importance values for the best-performing models. The *x*-axis of each graph represents the reduction in AUC when the feature was randomized in the testing samples. Positive error bars indicate the standard deviation of the averaged importance values. Each feature is shown in the same color in all bar graphs.

Machine learning can be used to train models to predict target variables, such as the mode of delivery in the present case, from unknown samples using input variables such as the relative abundances of bacteria. In the initial training of the ML models, we used four algorithms (RF, EXTRA, LGBM, and MLP) to differentiate between children born by vaginal delivery and Caesarean delivery, using the gut microbiome data from fecal samples obtained for each cohort at each of the time points (Fig. 4).

The ML models were, indeed, effective in predicting the mode of delivery on this basis at 1–2 months of age. The ML models achieved high AUC values ranging from 0.73 to 0.82 in all cohorts, depending on the ML model selected (Fig. 4), while the RF models were the best in the COPSAC (AUC = 0.73), NHBCS (AUC = 0.79), NOMIC (AUC = 0.82), and WHEALS (AUC = 0.79) cohorts. The EXTRA models performed well in the INFANTMET (AUC = 0.80) and JORVI (AUC = 0.74) cohorts, but the LGBM model achieved the highest AUC only in the HOUSTON cohort (AUC = 0.75). The MLP models achieved lower AUC values overall than the other models (Fig. 4B). PR curves can be found for all models in Fig. S2.

To control for the potentially confounding effect of breastfeeding, the same analyses were run separately in children receiving breastfeeding (Fig. S3). In COPSAC (AUC = 0.73) and INFANTMET (AUC = 0.75), the prediction performance remained the same or slightly lowered, while in HOUSTON (AUC = 0.62) and JORVI (0.63) cohorts, the prediction performance was much lower. There were only 29 children who were exclusively formula-fed at 1–2 months of age (Table S4), which did not allow separate ML analyses in this subgroup.

### *Bacteroides* was the most important genus for the performance of machine-learning models trained on taxa at 1–2 months of age

Next, we determined which features of the gut microbiome data were most important for the performance of the ML models—i.e., in differentiating between the modes of delivery at 1–2 months of age. We identified the most important features of the ML models using the permutation importance method.

The relative abundance of *Bacteroides* in the gut microbiome had the greatest impact on the prediction performance at 1–2 months of age (Fig. 4C). This may be assessed by evaluating the decrease in the AUC when the *Bacteroides* feature is removed from the model. This reduced the model performance in multiple cohorts: 0.06 AUC in COPSAC, 0.21 in HOUSTON, 0.16 in INFANTMET, 0.10 in JORVI, 0.18 in NHBCS, 0.1 in NOMIC, and 0.25 in WHEALS (Fig. 4C). Other important features for differentiating between the delivery modes based on the gut microbiome data were *Bifidobacterium*, *Enterococcus*, the *Escherichia-Shigella* complex, *Streptococcus*, *Veillonella*, and *Parabacteroides* (Fig. 4C).

### ML models were poor at accurately predicting the mode of delivery from microbiome taxonomic data recorded at 3–6 months or 9–12 months

**Fig 5:**
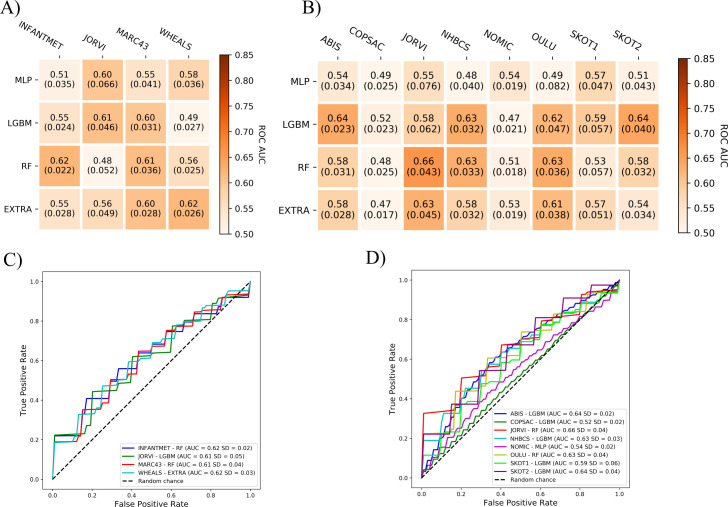
Performance of the ML model when predicting the mode of delivery from samples taken at 3–6 months or 9–12 months after birth. The MLP, LGBM, RF, and EXTRA models were trained independently to differentiate between vaginal delivery and Caesarean delivery samples using the relative abundances of gut bacteria at (**A**) 3–6 months and (**B**) 9–12 months after birth. ROC curves for the best-performing models were drawn for (**C**) 3–6 months and (**D**) 9–12 months after birth. The AUC values range between 0.5 and 1.0, where predictions from a model with a performance close to 0.5 would be equivalent to a random guess, and those from a model with a performance of 1.0 would always be correct.

When using gut microbiome taxonomic data from fecal samples taken at 3–6 months and 9–12 months of age, the ML models were not able to differentiate accurately between the modes of delivery of the children in any of the cohorts (Fig. 5; Fig. S4 and S5). The AUC of the best models ranged from 0.61 to 0.62 in four cohorts with gut microbiome data available at 3–6 months of age (Fig. 5A and C). Similarly, models trained using fecal samples collected 9–12 months after birth were unable to differentiate reliably between children born vaginally or via a Caesarean delivery (Fig. 5B and D).

### Machine-learning models failed to recognize the delivery mode using predicted metabolic pathway features at any infant age

Next, we used 16S rRNA gene sequences to infer the metabolic pathway composition of each sample with PICRUSt2, which can be used to generate an estimation of metabolic pathway composition based on the 16S rRNA amplicon sequencing data and a reference database. The performance of the ML models trained with predicted metabolic pathways in differentiating Caesarean delivery samples from vaginal delivery samples was comparable to that observed for the genera collapsed models in some cohorts, while in others, the performance values were much lower (Fig. S6). The ML models that used predicted metabolic pathways could not accurately predict the mode of delivery from gut microbiome samples collected 3–6 months or 9–12 months after birth, except in the Oulu (AUC = 0.72, SD = 0.03) and Jorvi (AUC = 0.75, SD = 0.07) cohorts at 9–12 months group (Fig. S7).

The ML models showed several metabolic pathways in the gut microbiome that were important for the performance of the models, such as carbohydrate degradation, nucleotide degradation, and fermentation of the pyruvate metabolic pathways. At 1–2 months of age, the ML models achieved an AUC of 0.7–0.8 for the COPSAC, NHBCS, and NOMIC cohorts, with the carbohydrate degradation pathway (PWY-7456) emerging as the most important performance feature (Fig. S6C).

The ML models for the INFANTMET cohort achieved moderate AUC scores, but unlike the other ML models in the three previously mentioned cohorts, they had pathways related to pyruvate fermentation and amino acid degradation as the top performance features. The best ML models in the HOUSTON, JORVI, and WHEALS cohorts, which all achieved low AUC scores (0.61–0.66), had a variety of pathways as their most important features (Fig. S6C).

The mean relative abundances and standard deviations of metabolic pathways can be found in the Supplementary Table (Table S6).

### Cross-study machine-learning models trained on taxonomy, but not on function, performed well when identifying the delivery mode at 1–2 months of infant age

**Fig 6:**
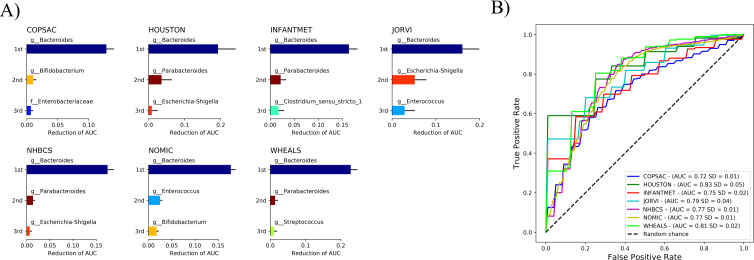
ML models were used to predict the mode of delivery when testing samples from other cohorts in a cross-study manner 1–2 months after birth. The mode of delivery was predicted for the test samples in each cohort by combining the best ML models from each of the other cohorts to form an ensemble classifier. The ML models had no previous knowledge of the other cohorts. (**A**) The permutation importance of the ensemble classifiers are visualized, with the *x*-axis of the graphs representing the reduction in the AUC when the feature is randomized in the test samples. Positive error bars were plotted to represent the standard deviation of the averaged importance values. Each feature is shown in the same color in all bar graphs. (**B**) ROC curves were drawn for the ensemble classifiers. The AUC values ranged between 0.5 and 1.0. Predictions from a model with a performance close to 0.5 are equivalent to a random guess, while a model with 1.0 is always correct.

The ML models trained with all the other cohorts and then tested on the remaining cohort performed well with all cohorts at 1–2 months after birth (Fig. 6), and the test samples from HOUSTON (AUC 0.83, SD 0.05), JORVI (AUC 0.79, SD 0.04), and WHEALS (AUC 0.81, SD 0.02) were predicted more accurately by the cross-study ML models than were those originally trained on the cohort’s own training samples (Fig. 6B). The cross-study ML models achieved fairly high accuracy when applied to the COPSAC (AUC 0.72, SD 0.01), INFANTMET (AUC 0.75, SD 0.02), NHBCS (AUC 0.78, SD 0.01), and NOMIC (AUC 0.77, SD 0.01) cohorts (Fig. 6B).

### *Bacteroides* was the most important feature when studying samples from other cohorts at 1–2 months after birth

Next, every feature was removed from the cross-study testing data one at a time by the permutation importance method; meanwhile, the average reduction of prediction performance was recorded for each feature. The most important feature when predicting the mode of delivery using gut microbiome data at 1–2 months of age was *Bacteroides* (Fig. 6A), while *Escherichia-Shigella*, *Parabacteroides*, *Bifidobacterium*, and *Enterococcus* had a lesser impact on the performance of the ML model. Removing *Bacteroides* from the testing data reduced the prediction capability of the model by more than half in every cohort.

### Bacteroides is relatively enriched in vaginally delivered infants at 1–2 months of age

**Fig 7:**
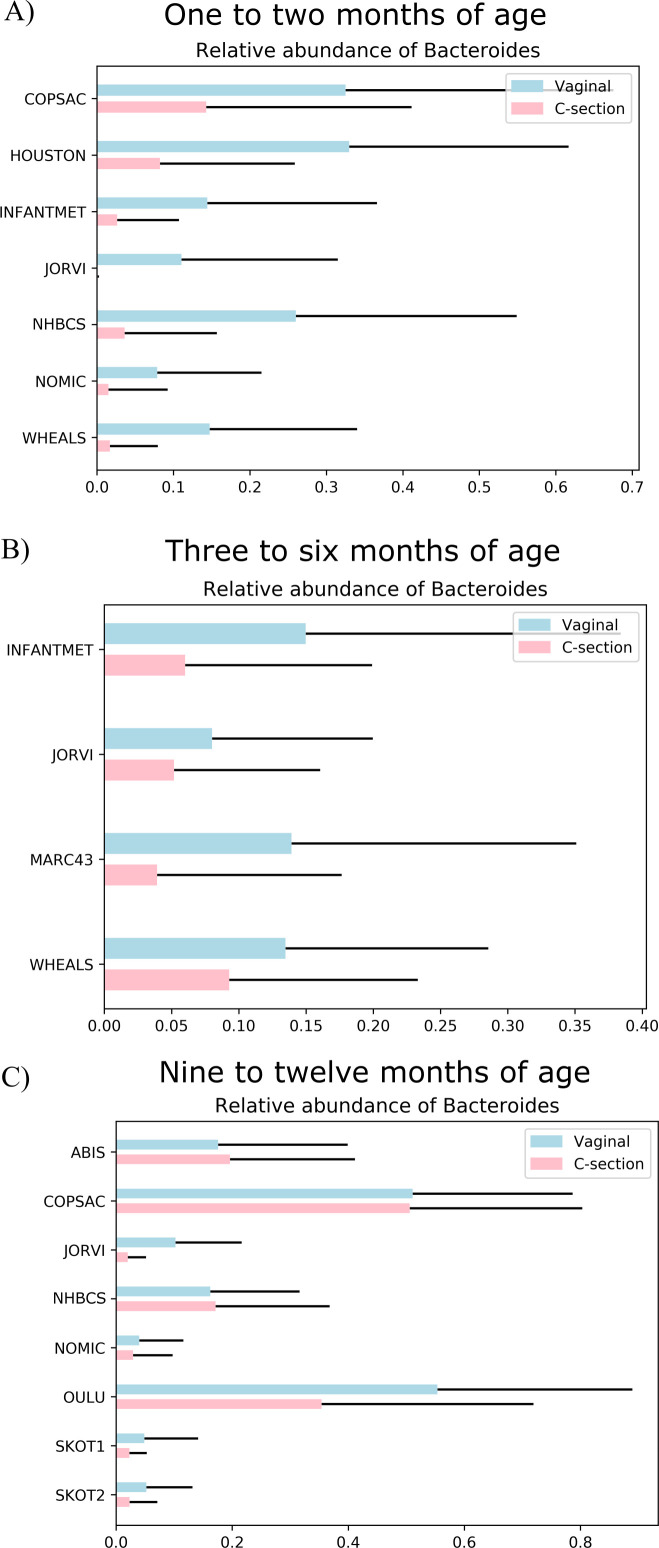
Combined mean relative abundances of sequences classified into the genus *Bacteroides* in each cohort. Mean relative abundance and standard deviation of *Bacteroides* in cohorts sampled approximately (**A**) 1–2 months, (**B**) 3–6 months, and (**C**) 9–12 months after birth, partitioned by mode of delivery. Light blue bars denote vaginally delivered infants, and pink bars denote those born by Caesarean delivery. The black lines are positive error bars (standard deviation).

To investigate why *Bacteroides* was shown as the most important feature in the ML models, we calculated the mean relative abundance of *Bacteroides* in each cohort in both the Caesarean delivery and vaginal delivery groups and plotted them side by side (Fig. 7). *Bacteroides* had a higher mean relative abundance in children born via Caesarean delivery in all cohorts at 1–2 months of age (Fig. 7A; Table S6), and similarly in all samples collected at roughly 3–6 months (Fig. 7B), while only a few cohorts had a higher mean relative abundance in vaginally delivered infants at 9–12 months after birth (Fig. 7C). The standard deviation for the relative abundance of *Bacteroides* was nevertheless very high at all three time points, indicating that the proportion of this genus differed greatly from one infant to another. The mean relative abundances and standard deviations of all genera shown as important by ML analyses can be found in Supplementary Table (Table S6).

### Alternative model building approaches did not improve the cross-study model performance at 1–2 months of age

Since there is no gold standard for building ML models or pre-processing microbiome data, we tested how a few different methods affected the cross-study predictions at 1–2 months. Alternative methods used were PipelineSearch and control augmenting. Using the genera-collapsed feature table to train the models and then combining all the other models into an ensemble voting classifier to predict the samples in one cohort was the strategy that performed best in all the cohorts except INFANTMET (Fig. S8). The Control augmenting (AUC = 0.80, SD = 0.045) and Pathway ensemble (AUC = 0.76, SD = 0.022) methods both achieved a higher AUC than the genera ensemble method (AUC = 0.75, SD = 0.018) when predicting delivery in the case of the INFANTMET samples. PipelineSearch did not have the highest AUC when predicting delivery mode in a cross-study way.

## DISCUSSION

We experimented with several ML model-building strategies to identify the best approach to combine and compare gut microbiome composition from 12 pediatric cohort studies. We chose mode of delivery as the exposure factor since it is well known that C-section delivery has an effect on infant gut microbiome. We showed that ML models, including MLP, LGBM, RF, and EXTRA models, were able to identify the mode of delivery of infants based on their gut microbiome taxonomic data at 1–2 months of age. *Bacteroides*, enriched in the gut microbiome of infants born by the vaginal route, was the most important feature in the ML models for identifying the mode of delivery. When the infants were older, all ML models performed poorly. Similarly, all ML models performed poorly when trained on predicted microbiome function at all ages.

In the present study, we used ML analysis of gut microbiome composition retrieved from high-quality prospective cohorts from Europe and USA in predicting the mode of delivery. Previously, Le Goallec et al. aggregated 1,570 samples from 300 infants included in 4 European studies to form a single data set from the first 3 years of life (18). Their models were used to predict host characteristics, such as age, sex, country of origin, antibiotic usage, delivery mode, and breastfeeding status. In their study, adding microbiome data to the model increased the prediction performance from an AUC of 0.59 to 0.76 as compared to demographic factors alone in predicting delivery mode (18). Here, we show that based on microbiome taxonomic data alone, cross-study ML models were able to predict delivery mode accurately in infants under 3 months of age with AUC ranging from 0.72 to 0.83 depending on the cohort and the algorithm used. However, when using gut microbiome data from fecal samples taken at 3–6 months and 9–12 months of age, or alternately training on the predicted functional metabolic pathways at any age, the ML models were not able to differentiate accurately between the modes of delivery of the children in any of the cohorts.

There are only a few other earlier studies of cross-study ML in gut microbiome research (17, 20, 54). In a study investigating the role of the gut microbiome in patients with type 2 diabetes, random forest models trained on cross-study data were able to predict type 2 diabetes status (17). In another study examining gut microbiome composition in adult obesity with a cross-study design using 10 data sets, the median accuracy of the ML analyses in distinguishing obesity based on the gut microbiome data was close to that of a random chance classifier (54). In a large study using a cross-disease design, ML models trained to predict one disease lost their accuracy when naively transferred to predict samples from other disease data sets (20). The authors of the study, however, suggested a method called “control augmenting,” in which control samples from outside cohorts are added to the training data to increase portability between data sets by the data augmenting method.

The subsequent health of children after Caesarean delivery has been reported in several previous epidemiological observational studies, associating with asthma (1, 2), food allergies (3, 4), obesity (5), and diabetes (6). Furthermore, Caesarean delivery has been associated with alterations in the gut microbiome composition in infants, with the relative abundance of *Bacteroides* (9–14), *Escherichia-Shigella* (9), and *Parabacteroide*s (10) being lower than in vaginal deliveries. Similarly, we found that *Bacteroides* was the most important taxonomic feature when predicting the mode of delivery of infants based on the gut microbiome at 1–2 months in all cohorts studied here. In addition, we found that the mean relative abundance of *Bacteroides* was lower at 1–2 months of age in the Caesarean delivery infants than in those born by vaginal delivery. When using the predicted metabolic pathways of the gut microbiome, the carbohydrate degradation pathways were of greater relative importance in classifying by mode of delivery. However, no pathway could consistently predict mode of delivery at any gestational age which is similar to the results previously reported by Chu et al. (27). Caesarean deliveries, performed for multiple underlying maternal and fetal indications, are associated with varying rates of success at exclusive breastfeeding (55). In the present study, we planned to perform a sensitivity analysis stratified on feeding mode. However, due to low number of infants who received exclusive formula feeding, among those in whom we had information on mode of feeding, we were not able to perform this analysis.

Previous microbiome studies have used various algorithms, such as random forest (17, 18, 20), support vector machine (17, 18), elastic net (17, 18), and gradient boosted machine (18, 56). Decision tree-based algorithms such as random forest and LGBM have consistently been among the top performers in studies employing multiple data sets (17, 18, 20). Additionally, deep learning approaches have shown promise (49), and consequently, we selected three decision tree-based algorithms and one neural network for our analyses. Our results suggest that even relatively similar decision tree algorithms perform differently in each data set, so each algorithm needs to be validated on a data set-by-data set basis. Interestingly, multilayer perceptron performed poorly relative to the decision tree-based algorithms in our study.

Pre-processing choices made before training the ML models affected the downstream analyses. The choice of a taxonomic database, the quality filtering parameters, and collapsing to a specific taxonomic level are all likely to affect the downstream ML analyses. We, therefore, tested four model-building approaches using the same cross-validation folds for each method. As a baseline, we built models on the genera (SILVA [39] database) collapsed feature tables. Second, we built models based on predicted metabolic pathway feature tables, and the third alternative method was to use independent control samples to augment each cohort, as presented in a previous study (20). Lastly, we used a novel method called “PipelineSearch,” in which each model could select data from various pre-processing routes—e.g., Greengenes instead of SILVA as a taxonomic database and predicted metabolic pathway features instead of genera-collapsed features. Interestingly, the PipelineSearch method could not achieve the same prediction performance as the baseline genera-collapsed models even though the models could select the same data to be used. This could be explained by the volatility of the microbiome data and the relatively low number of available samples in each cohort. Nevertheless, PipelineSearch is useful in cases where researchers lack the necessary domain knowledge to make optimal pre-processing choices; instead, they can supply the PipelineSearch model with a variety of methods even though some of those pre-processing choices may be suboptimal.

The present study has several strengths. The use of ML in gut microbiome analysis in a cross-study way, although it has been employed in previous studies (17, 20, 54), is still a novel approach. We had gut microbiome data from 4,099 samples representing 12 infant cohorts in their first year of life, and by using ML models, we were able to show predictable differences on the composition of the gut microbiome appears to have certain universal characteristics in cohorts of 1- to 2-month-old infants born by Caesarean delivery across populations. However, this was limited to relative abundance differences in a single taxa, Bacteroides, and was not accompanied by changes in the predicted functional metagenome. Furthermore, the use of active data sharing and collaboration enabled the analysis of a varied collection of data sets spanning Europe and the United States. Finally, we have successfully combined two research fields: clinical medicine and computational biology.

Nevertheless, there are some limitations to our study. To assign taxonomy, we used the SILVA database (version 138). *Bacteroides* has been reclassified as *Phocaeicola* (57); consequently, the genera names shown here may change in the future release of SILVA database. ML analyses do not allow for direct controlling for various factors. We did, however, perform ML analyses separately in subgroups depending on breastfeeding status. We used the PICRUSt2 bioinformatic tool to predict microbial metabolic pathway composition data, which might not correspond to the actual metagenomic data produced by whole-genome sequencing. Furthermore, the study results are generalizable to term infants because we excluded cohorts with mainly preterm infants. Finally, the cohorts recruited for this study were not created solely to study the effects of the mode of delivery. As such, the cohort structures and designs varied from one cohort to another. Furthermore, we were not able to investigate emergency C-section and elective C-section groups separately due to low sample sizes and lack of required data.

Our study provides a new perspective on microbiome research, as it shows that ML enables data analyses in gut microbiome research by comparing and combining data sets from multiple cohorts collected in different countries across diverse patient populations. Furthermore, there is a crucial need to shift the research paradigm from merely retrospective predictions to a more proactive approach, where extensive investigation is directed toward anticipating the health outcomes of infants and children through the analysis of the gut microbiome. This proactive stance could provide a deeper understanding of how the gut microbiome influences the well-being of infants and children and potentially lead to more effective strategies for promoting their optimal health and development.

## AFTER EPUB

*[This article was published on 24 October 2023 with typographical errors in the introduction and Materials and Methods. The text was corrected in the current version, posted on 30 October 2023.]*

## SUPPLEMENTAL MATERIAL

Data S1 msystems.00364-23-s0001.xlsx Genera collapsed relative abundance feature tables of each cohort used in ML analyses.

Data S2 msystems.00364-23-s0002.xlsx Predicted metabolic pathway relative abundance feature tables used in ML analyses at 1-2 months.

Data S3 msystems.00364-23-s0003.xlsx Predicted metabolic pathway relative abundance feature tables used in ML analyses at 3-6 months.

Data S4 msystems.00364-23-s0004.xlsx Predicted metabolic pathway relative abundance feature tables used in ML analyses at 9-12 months.

Supplemental Information msystems.00364-23-s0005.docx Figures S1 to S8.

Supplemental Tables msystems.00364-23-s0006.xlsx Tables S1 to s6.

ASM does not own the copyrights to Supplemental Material that may be linked to, or accessed through, an article. The authors have granted ASM a non-exclusive, world-wide license to publish the Supplemental Material files. Please contact the corresponding author directly for reuse.
