# Supplementary material for: Machine-learning analysis of cross-study samples according to the gut microbiome in 12 infant cohorts
Source: mSystems. 2023 Oct 24;8(6):e00364-23. doi: 10.1128/msystems.00364-23 (PMC10734493; doi:10.1128/msystems.00364-23)
Supplement: Supplemental Information — Figures S1 to S8. [file msystems.00364-23-s0005.docx]

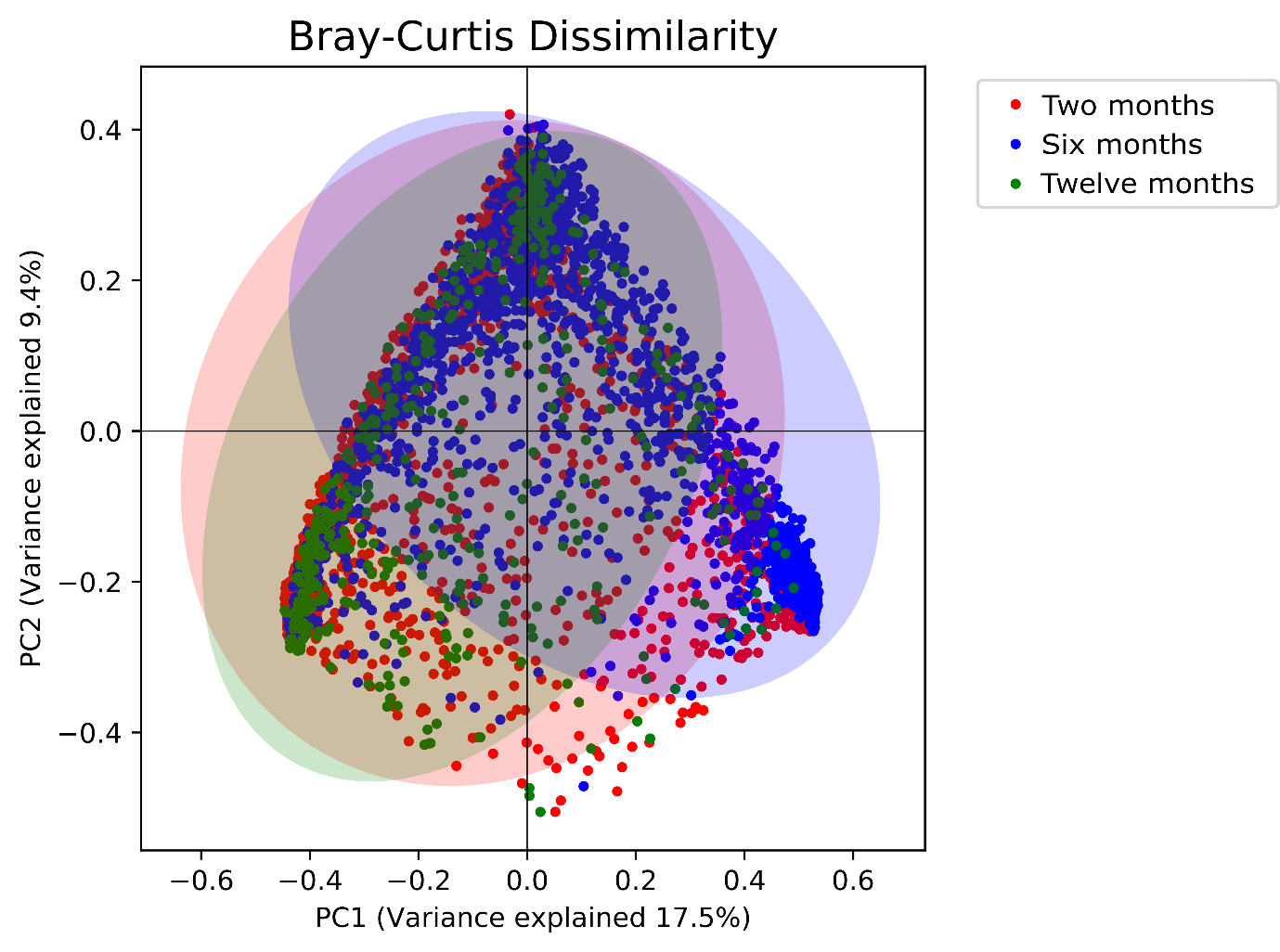


**Supplementary Fig 1. Beta diversity indices clustered for each sampling time point.** Between-sample diversity was analysed according to sampling time using Bray-Curtis dissimilarity using principal coordinate analysis (PCoA). The samples from each time point (1–2 months, 3–6 months, and 9–12 months) were drawn in a different colour and the confidence ellipse was drawn using the Pearson correlation coefficient for each cohort.


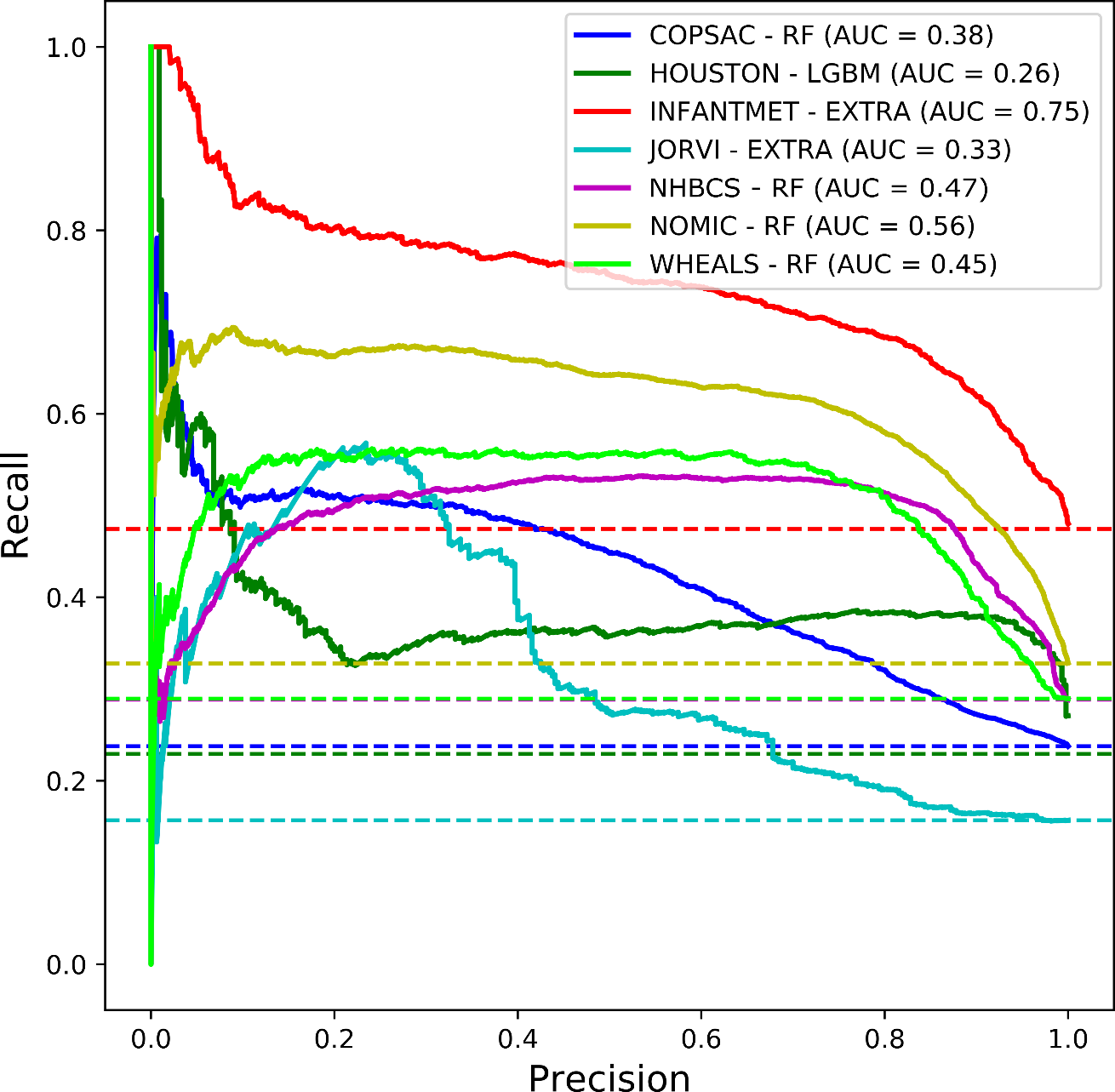


**Supplementary Fig. 2. Precision-recall curve of ML models when predicting delivery mode of children based on gut microbiome at 1–2 months of age.** Caesarean section was assigned as the positive class while vaginal delivery was the negative class. Precision, the proportion of correctly predicted positives from all positive predictions, is plotted on the x-axis. Recall, the proportion of correctly predicted positives predictions from all positive samples, is plotted on the y-axis. The precision-recall curve of a random chance classifier is plotted for each cohort as the dotted horizontal line.


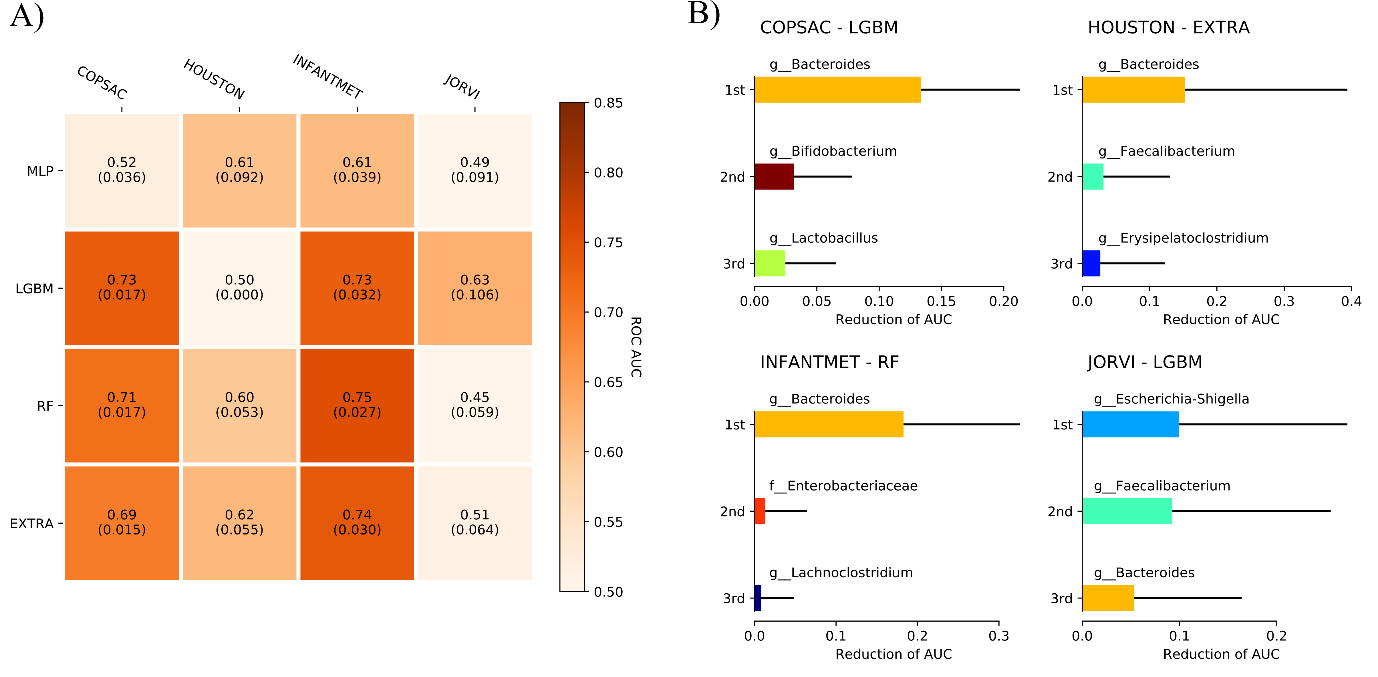


**Supplementary Fig. 3. Performance and feature importance’s for ML models predicting the mode of delivery using the gut microbiome in the first 2 months of age, while exclusively formula fed samples were removed.** The MLP, LGBM, RF and EXTRA models were trained independently to differentiate between vaginal delivery and Cesarean delivery samples using the relative abundances of taxa in the gut microbiome sampled at one- to two-months after birth. Receiver Operating Characteristic curves Area-under-the-curve (ROC AUC) values ranged between 0.5 and 1.0. Predictions from a model with a performance close to 0.5 are equivalent to a random guess, whereas a model with 1.0 is always correct. **B)** The permutation importance’s of the best-performing models are visualized, with the x-axis of the graphs representing the reduction in AUC when a given feature is randomized in the testing samples. Positive error bars were plotted to represent the standard deviation of the averaged importance values. Each feature is shown in the same colour in all the bar graphs.


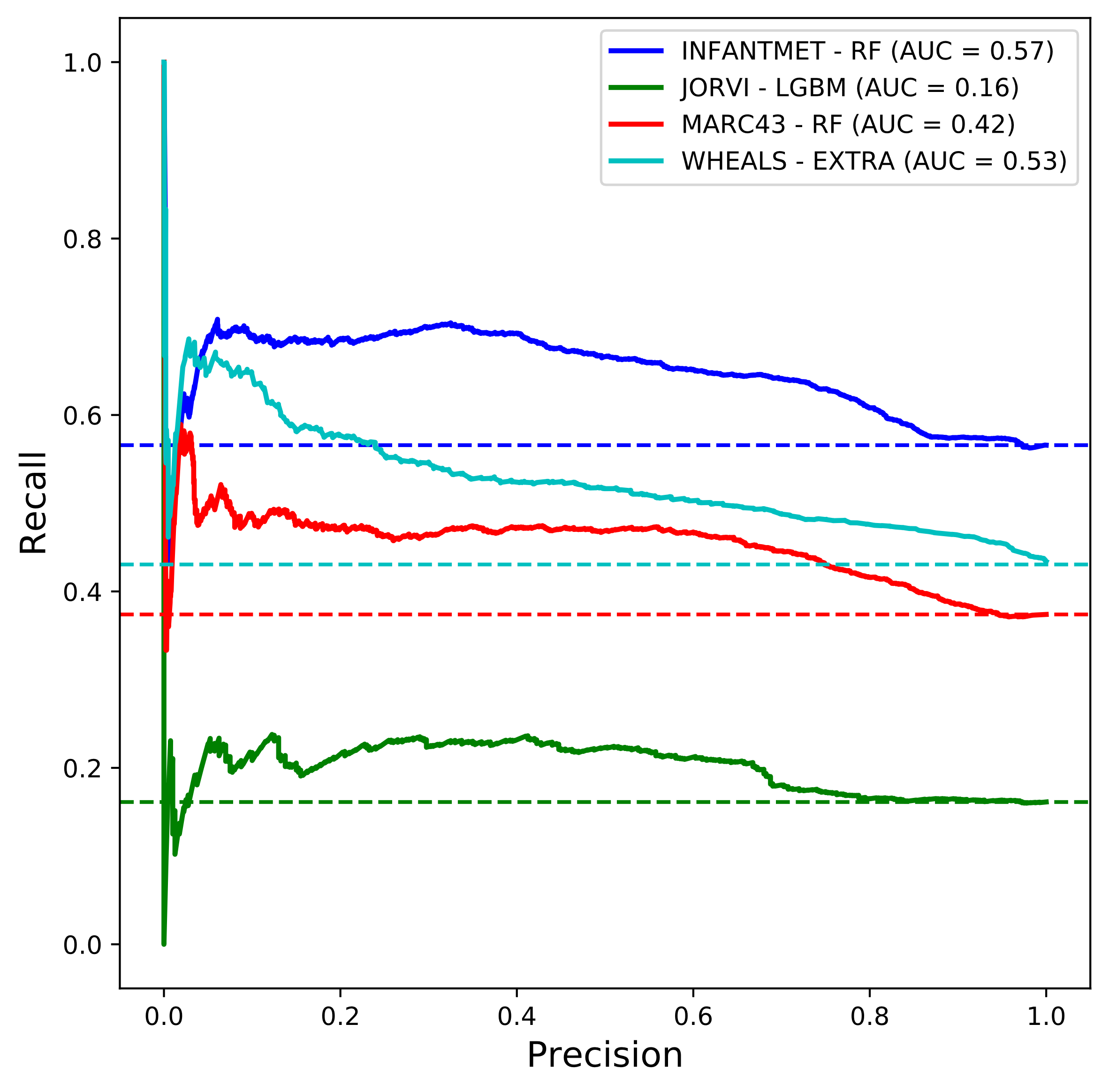


**Supplementary Fig. 4. Precision-recall curve of ML models when predicting delivery mode of children based on gut microbiome at 3–6 months of age.** Caesarean section was assigned as the positive class while vaginal delivery was the negative class. Precision, the proportion of correctly predicted positives from all positive predictions, is plotted on the x-axis. Recall, the proportion of correctly predicted positives predictions from all positive samples, is plotted on the y-axis. The precision-recall curve of a random chance classifier is plotted for each cohort as the dotted horizontal line.


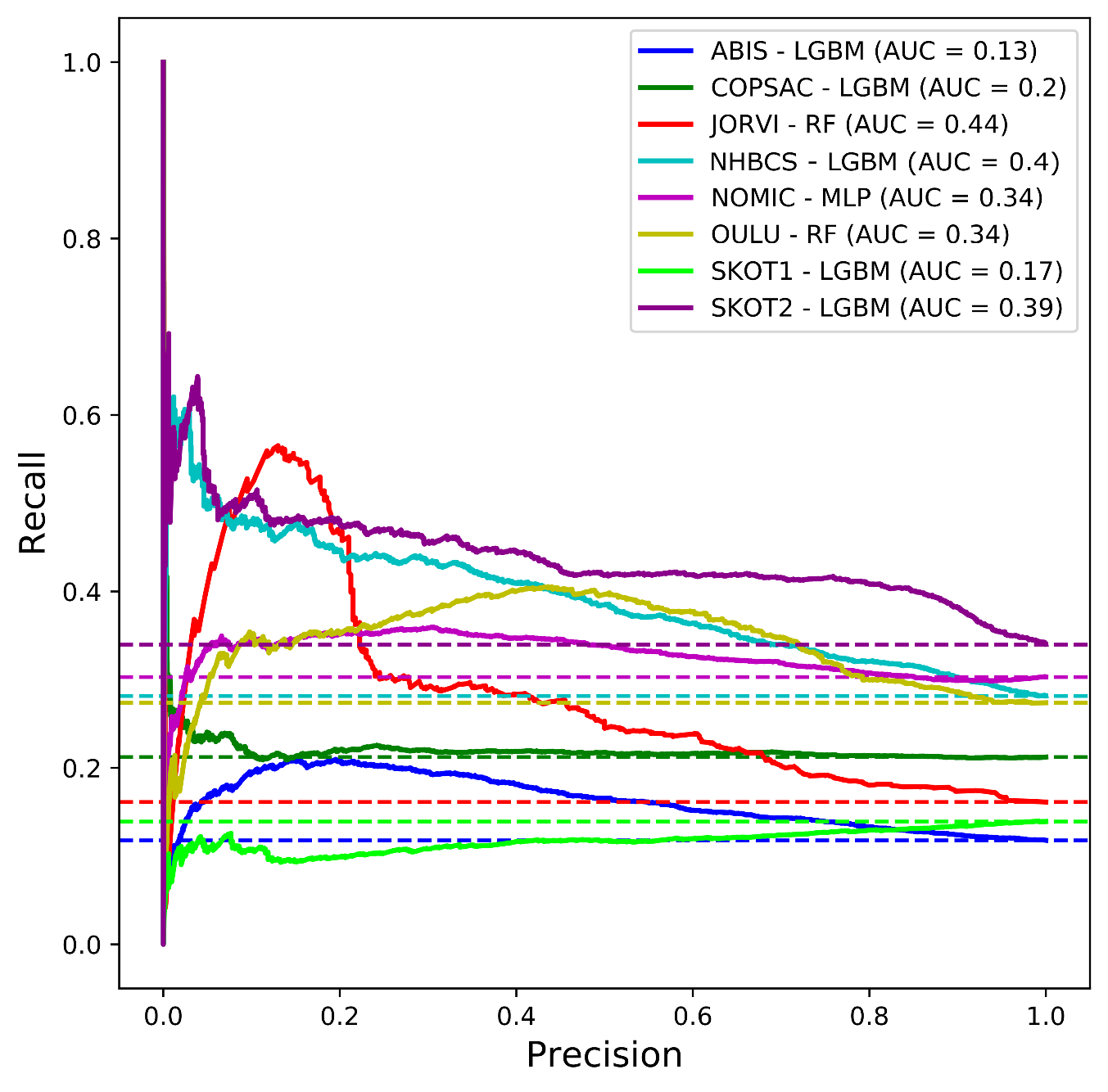


**Supplementary Fig. 5. Precision-recall curve of ML models when predicting delivery mode of children based on gut microbiome at 9–12 months of age.** Caesarean section was assigned as the positive class while vaginal delivery was the negative class. Precision, the proportion of correctly predicted positives from all positive predictions, is plotted on the x-axis. Recall, the proportion of correctly predicted positives predictions from all positive samples, is plotted on the y-axis. The precision-recall curve of a random chance classifier is plotted for each cohort as the dotted horizontal line.


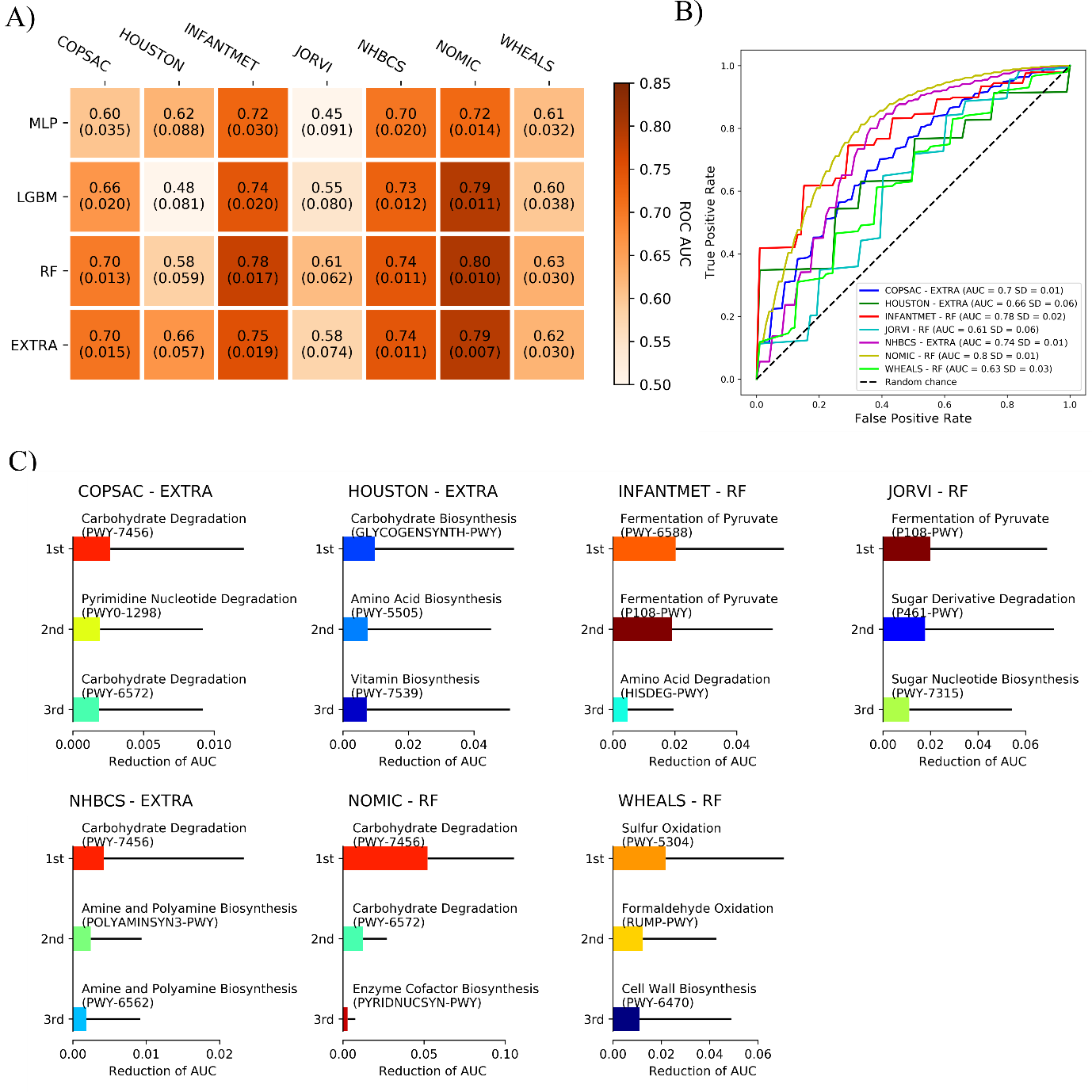


**Supplementary Fig. 6. ML models differentiate between vaginal and Caesarean delivery using predicted metabolic pathways in the gut microbiome of infants based on samples obtained approximately 1–2 months after birth.** The delivery modes were either vaginal delivery or Caesarean delivery, and the ML models used the gut microbiomes of the infants as assessed from faecal samples obtained approximately 1–2 months after birth in seven infant cohorts. **A)** Best performances of the MLP, LGBM, RF, and EXTRA models, trained independently to differentiate between vaginal delivery and Caesarean delivery samples using the relative abundances of predicted metabolic pathways of the microbiome at 1–2 months after birth, shown separately for each cohort. **B)** Receiver- operating-characteristic (ROC) curves for the best-performing models. The area under the curve (AUC) values for these ROC curves indicate model performances that range between 0.5 and 1.0. Predictions from a model with a performance close to 0.5 are equivalent to a random choice, whereas a model with an AUC of 1.0 would hypothetically be a perfect model and classify all children correctly. **C)**Permutation importance values for the best-performing models. The x-axis of each graph represents the reduction in AUC when the feature was randomized in the testing samples. Positive error bars indicate the standard deviation of the averaged importance values. Each feature is shown in the same colour in all bar graphs.


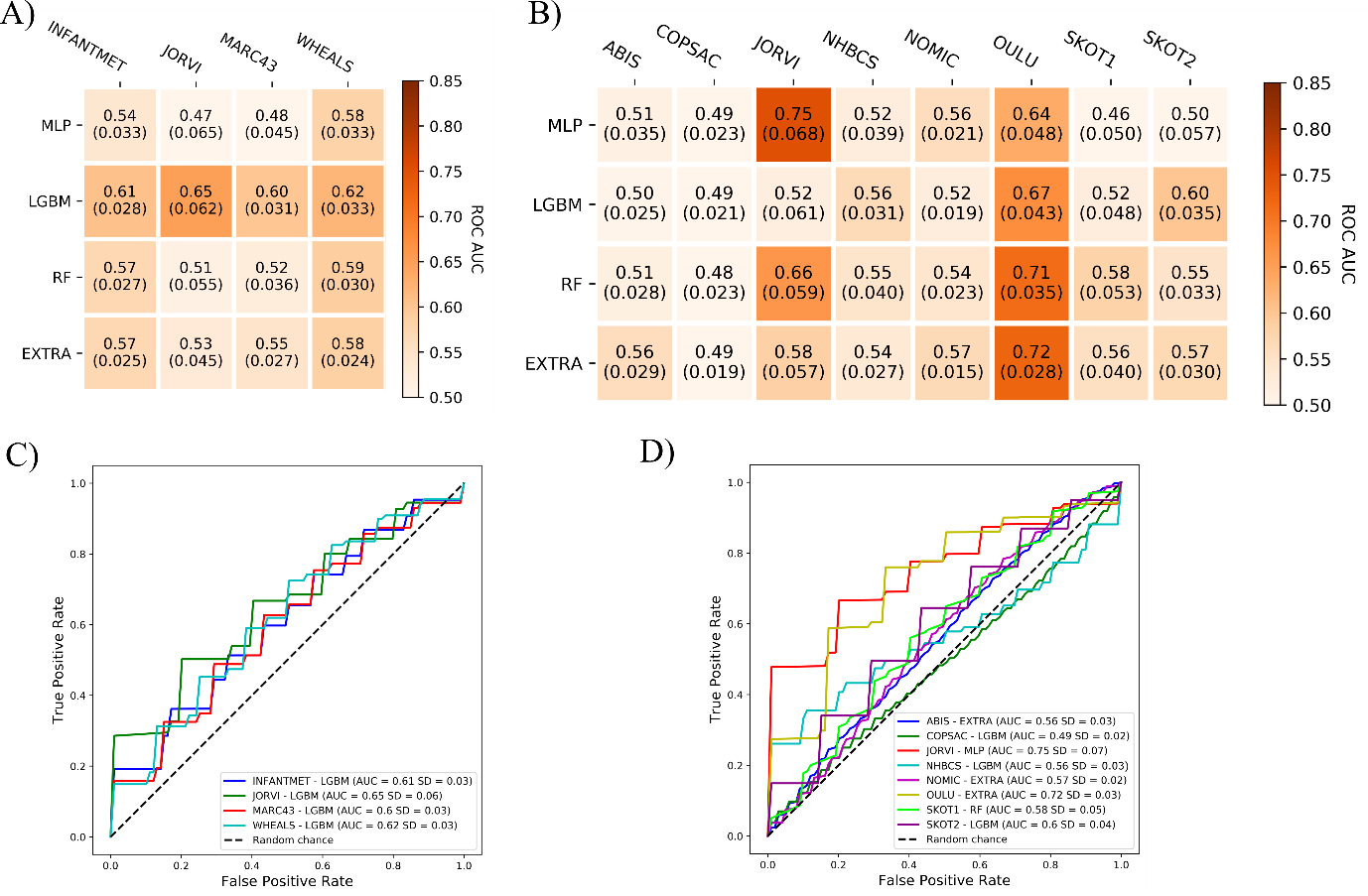


**Supplementary Fig. 7. Performance values for ML models predicting the mode of delivery using predicted metabolic pathways sampled at 3–6 months and one year after birth. The** MLP, LGBM, RF and EXTRA models were trained independently to differentiate between vaginal delivery and Caesarean delivery samples using the relative abundances of predicted metabolic pathways in the gut microbiome at **A)** 3­–6 months and **B)** 9–12 after birth. Receiver Operating Characteristic curves for the best-performing models were drawn for **C)** 3–6 months and **D)** 9–12 months after birth. The area under the curve (AUC) values range between 0.5 and 1.0, where predictions from a model with a performance close to 0.5 would be equivalent to a random guess, and those from a model with a performance of 1.0 would always be correct.


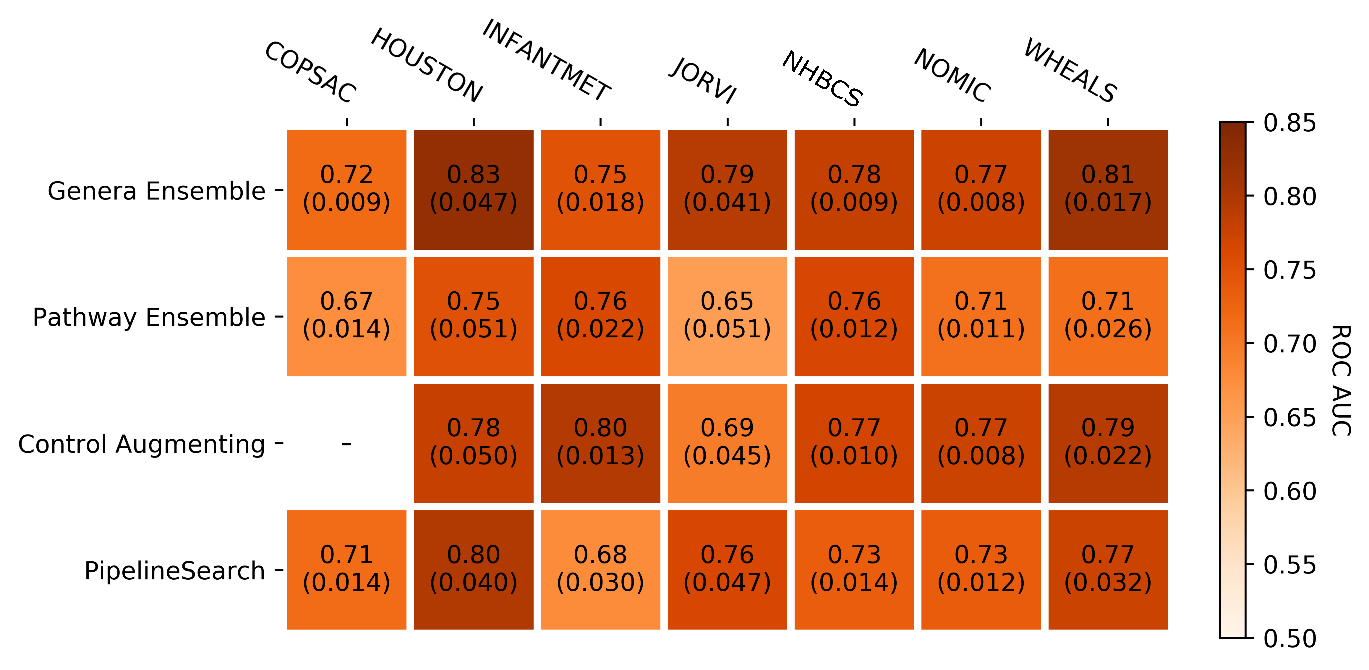


**Supplementary Fig. 8. Four methods were used to predict the mode of delivery from the other cohorts when testing samples taken 1-2 months after birth in a cross-study manner.** The modes of delivery of the test samples were predicted in each cohort by combining the best ML models from each of the other cohorts into an ensemble classifier, using genera data to train the models ion the Genera ensemble method, predicted metabolic pathways produced with PICRUSt2 in the Pathway ensemble method and genera data with additional control samples from the COPSAC cohort added to the training data in the Control augmenting method. PipelineSearch models were given the option of choosing either SILVA or Greengenes-based data, together with genera or predicted pathway data during parameter tuning. The ML models had no previous knowledge of the other cohorts.
